# Assessment of deep learning and transfer learning for cancer prediction based on gene expression data

**DOI:** 10.1186/s12859-022-04807-7

**Published:** 2022-07-03

**Authors:** Blaise Hanczar, Victoria Bourgeais, Farida Zehraoui

**Affiliations:** IBISC, Université Paris-Saclay (Univ. Evry), 23 boulevard de France, 91034 Evry, France

**Keywords:** Deep neural network, Transfer learning, Phenotype prediction, Gene expression

## Abstract

**Background:**

Machine learning is now a standard tool for cancer prediction based on gene expression data. However, deep learning is still new for this task, and there is no clear consensus about its performance and utility. Few experimental works have evaluated deep neural networks and compared them with state-of-the-art machine learning. Moreover, their conclusions are not consistent.

**Results:**

We extensively evaluate the deep learning approach on 22 cancer prediction tasks based on gene expression data. We measure the impact of the main hyper-parameters and compare the performances of neural networks with the state-of-the-art. We also investigate the effectiveness of several transfer learning schemes in different experimental setups.

**Conclusion:**

Based on our experimentations, we provide several recommendations to optimize the construction and training of a neural network model. We show that neural networks outperform the state-of-the-art methods only for very large training set size. For a small training set, we show that transfer learning is possible and may strongly improve the model performance in some cases.

## Background

The transcriptomics technologies (microarray, RNA sequencing) provide massive molecular-scale information about the patients. The analysis of these gene expression profiles is one of the main challenges for the development of new tools for personalized medicine [1] and especially in oncology [2]. The analysis of these data may support the physician for the diagnosis of cancer, the classification of tumors, the outcome prognosis, and the individualized treatment decision. Complex pathologies, like cancers, disrupt gene expression, leaving signatures that can contain valuable information. The problem is that these signatures are complex non-linear combinations of genes hidden in the multiple gene expressions. Machine learning is the main approach to identify these signatures and to construct models making predictions from gene expression profiles. Many classical methods of the machine learning community have been adapted and tested in the transcriptomic context; this includes linear and quadratic models, support vector machines (SVM), random forest (RF), and boosting [3]. Although these methods produced promising results, constructing models that are accurate and robust enough for practical medical application is still an open problem. The most challenging problems are the high dimensionality of the gene expression data, the insufficient number of training examples that lead to overfitting during training, and lack of robustness of the results.

In the last ten years, deep learning has become one of the most important breakthroughs in machine learning [4]. Its primary application domain is image recognition and speech recognition, where it has beaten other machine-learning techniques. However, it is also promising in many other domains, particularly the biomedical sciences. Deep learning techniques have recently drawn attention in bioinformatics because of their automatic capturing of nonlinear relationships from their input and a flexible model design. However, deep learning methods are still very new in gene expression analysis, and few works have been published compared to the other machine learning methods [5].

Unlike images or text data, gene expression data has no structure that can be exploited in a neural network architecture. Therefore, the main architecture used for prediction from gene expression data is the multilayer perceptron (MLP). Fakoor et al. propose one of the first works to apply MLP on gene expression data to predict the presence of cancer or the sub-type of cancer [6]. Several works try to apply MLP to others types of prediction or use variants to improve the performances. Lai et al. use a multi-modal MLP to integrate clinical data with gene expression and predict the prognostic for non small cell lung cancer [7]. Chen et al. add to the classical cross-entropy used in the MLP, a clustering loss to the last hidden layer. Its purpose is to maximize the marge between each class in the latent space defined in hidden layers and provide better prediction of cancers [8]. In DeepSurv, Katzman et al. replace the classical output layer of a MLP by a Cox proportional hazard layer for modeling interactions between expression profile and treatment effectiveness [9]. Bourgeais et al. use a MLP whose architecture mimics the Gene Ontology to detect cancers and produce an explanation of the prediction [10].

Other types of architecture have been tested, like convolutional neural networks (CNN) or graph neural networks (GNN). However, they are facing the problem of lack of structure in the gene expression data. Mostafa et al. use CNN to predict tumor type from 1D or 2D expression profiles [11]. The groups of genes analyzed in convolution windows depend on the arrangement of the genes in the 1D-vector or the 2D-matrix. Unlike image data, this arrangement is random and does not represent specific information. Some works tried to integrate external information to identify a structure in the gene expression that CNN or GNN can exploit. For example, the co-expression networks or protein-protein interaction networks have been used to represent the gene expression profile by a graph, and the predictions are computed through a graph neural network [12, 13]. However, there is no consensus that this integration of structural information may help the network to extract relevant expression patterns and improve the prediction performance [14].

Transfer learning is often proposed to tackle the problem of small-training set and the high dimension of the gene expression data. The term transfer learning refers to a set of techniques to transfer information from a model (source) to another one (target). Transfer learning is widely used in image analysis and natural language processing, where some common visual or textual patterns are helpful for any classification task. They can be extracted from a large source dataset and transferred to the target dataset for the classification task.

There are different scenarios of transfer learning [15]. We can distinguish supervised transfer learning from unsupervised transfer learning. In supervised transfer learning, the labels of samples are used to build the source model, and we consider that the source classification task is close to the target classification task. Unsupervised transfer learning takes advantage of a large set of unlabeled source data to learn a new representation of the data. It generally consists of learning an encoder that projects the data into a small dimension space. In this case, we consider that the new data representation learned from source data, will be helpful for the target classification task. For the different scenarios, after the source model has been trained, the parameters a re copied to the target model. Note that it is possible to retrain the whole target model on target data; in this case, transfer learning is used as an initialization of the target model.

In the gene expression context, the standard approach is the unsupervised pre-training of the hidden layers of the MLP. This approach generally involves an autoencoder (AE) that compresses the gene expression profile into a small vector. Since the training of the AE is unsupervised, we can benefit from much larger datasets. Kim et al. train a variational autoencoder (VAE) from the pan-cancer TCGA dataset, then the hidden layers of the encoder are copied to the hidden layers of the MLP that predicts the hazard ratio of patient with a specific cancer [16]. In [17], each hidden layer of a MLP for cancer prognostic prediction is initialized with a denoising auto-encoder (DAE) trained from a large pan-cancer dataset. Alzubaidi et al. pre-train their MLP with a sparse auto-encoder to predict cancer subtypes and identify biomarkers [18].

There is no clear consensus about the performances and utility of deep learning for prediction tasks based on gene expression data. Few experimental works have been done to evaluate NN and compare them with state-of-the-art machine learning models. Moreover, their conclusions are not consistent. Indeed, Yu et al. show that shallow MLP is more accurate and robust than deep architecture, CNN, and classical machine learning methods for disease prediction [14]. Smith et al. evaluate the deep representation methods used in unsupervised pre-training, for cancer diagnosis, cancer stage, and survival prediction. They conclude that deep learning methods are not superior to the classical machine learning approaches [19].

The difficulty of obtaining reliable results about the performance of deep learning methods comes mainly from the large number of hyper-parameters involved in these approaches. Many parameters must be chosen to set the architectures of the NN, the learning algorithms, and the regularization techniques. Using a non-optimal value in one of these parameters may strongly affect the model’s performance.

Non-expert users may overlook the sensitivity of NN to the hyper-parameter values due to the increasing complexity of DNN models that make the parameter tuning task very hard. This leads these users to keep the default settings when training their models leading to sub-optimal results. Moreover, since a lot of pre-trained NN models are available, transfer learning is used without addressing all the strategies that can significantly improve the NN results. In this paper, we address these points by performing an exhaustive evaluation of MLP for several cancer prediction tasks based on gene expression data (microarray and RNA-Seq data). We measure the impact of the main hyper-parameters on the performance of NN and compare the NN with the state-of-the-art machine learning models (SVM, XGBoost, LASSO, RF). In addition, we investigate the usefulness of different transfer learning strategies, including unsupervised pre-training. To our knowledge, this is the first work that promotes the appropriate use of deep learning and transfer learning for biomedical prediction tasks and the most extensive experimental study that addresses this topic. Indeed, we trained and evaluated around 93,000 NN for performing 22 prediction tasks.

## Results

**Fig. 1:**
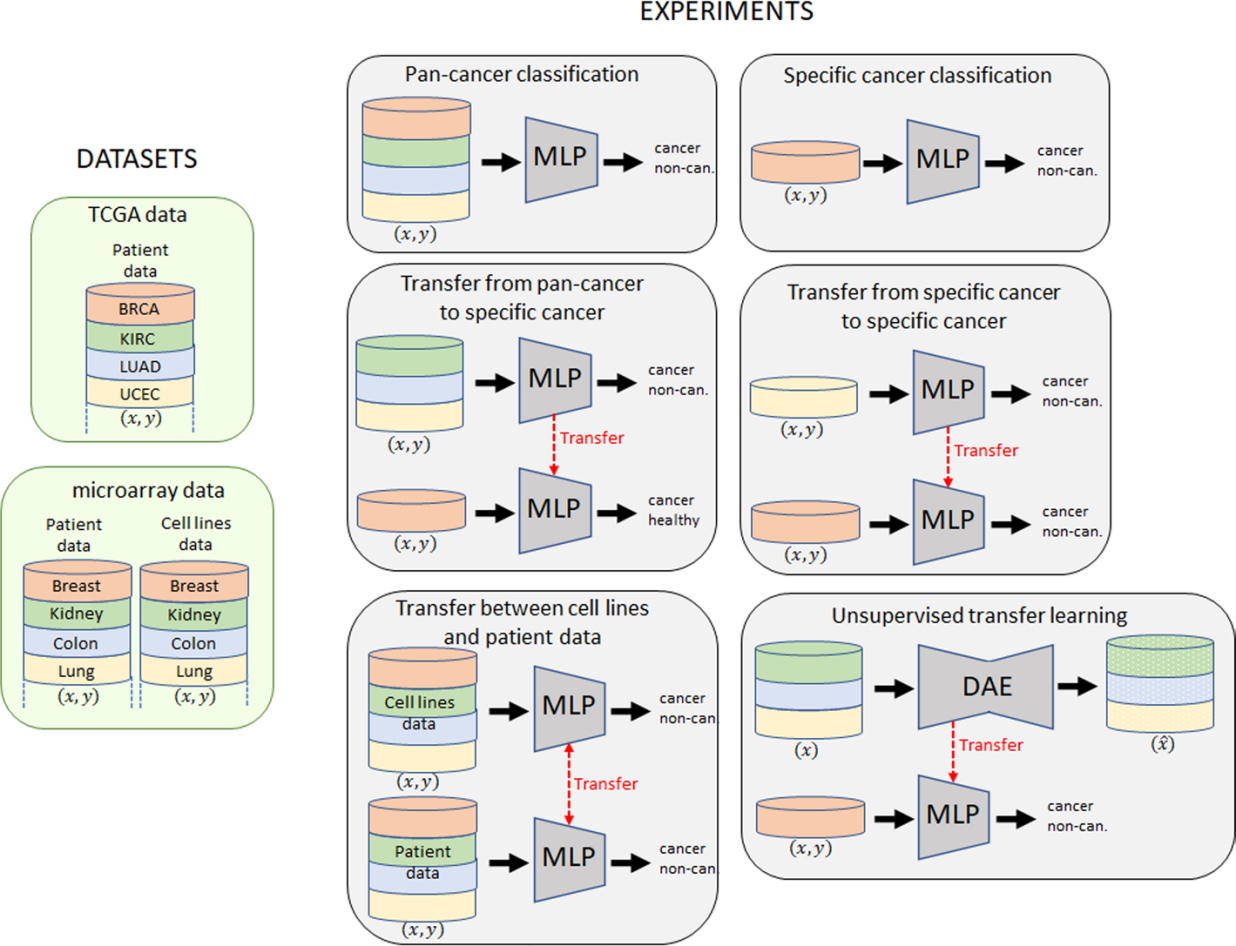
All experiments performed in this study

Two large gene expression datasets are used in our experiments. We tested the NN for pan-cancer and specific cancer classification, supervised transfer learning (from pan cancer to a specific cancer, from a specific cancer to another specific cancer, between cell lines and patient data) and unsupervised transfer learning. Figure 1 summarizes all these experiments.

### Data and experiment design

Our experimentation is based on two large datasets, including 22 classification tasks. The first one comes from a pan-cancer study of cross-experiments compiling the gene expression profiles from about 40,000 publicly available Affymetrix HG-U133Plus2 arrays [20]. It combines different gene expression datasets containing diverse tissues and experimental protocols and integrates both patient and cell line data. The dataset is accessible via the ArrayExpress database (accession number E-MTAB-3732). After quality control and normalization, the dataset contains the expression of 54,675 genes. The samples whose status is not clearly defined are dropped. We only keep samples from the most present tissues in the dataset (tissues with more than 400 samples). For each sample, the available information is its status (cancer/non-cancer), type (patient/cell line), and tissue. 12 classification tasks are associated with this dataset. It consists of predicting the presence of cancer in the pan-cancer case or in each of the 11 specific cancers. Note that the classes are unbalanced since cancer samples are three times more present than non-cancer samples. Table 1 gives the characteristics of this dataset. This dataset is divided into a training set of 13,000 samples and a test set of 3709 samples preserving the proportion of cancer/non-cancer samples.

**Table 1:** Characteristics of the microarray dataset

Disease	Size	Patients	Cell lines	Cancer	Non-cancer	Prior
Leukemias	4283	3452	831	2336	1947	0.55
Bone marrow cancer	3525	3374	151	3185	340	0.90
Breast cancer	2171	1366	805	1863	308	0.86
Kidney cancer	657	423	234	400	257	0.61
Liver cancer	727	312	415	601	126	0.82
Lung cancer	1415	749	666	818	597	0.58
Skin cancer	835	554	281	454	381	0.54
Brain cancer	869	468	401	819	50	0.94
Colon cancer	1239	875	364	1112	127	0.90
Ovary cancer	573	427	146	533	40	0.93
Prostate cancer	415	182	233	350	65	0.84
Total	16,709	12,182	4527	12,471	4238	0.75

The columns represent respectively the type of tissues (Disease), the numbers of samples (Size), patient samples (Patients), cell line samples (Cell lines), cancer samples (Cancer), non-cancer samples (Non-cancer) and the proportion of the majority class (Prior)

The second dataset comes from the TCGA portal,^1^ a repository of multi-omics datasets containing only real patients with several types of cancer [21]. We use the RNA-seq datasets containing at least 350 samples. This dataset is much more homogeneous than the first dataset, and many clinical annotations are available. We investigate two classification tasks in a pan-cancer context. The first one is the prediction of the presence of cancer, where the classes are very unbalanced since 92.7% of samples are labeled cancer. The second pan-cancer task is the prediction of the type of cancer. We also consider eight specific cancer tasks for the prediction of the presence of cancer. LGG, OV, and LIHC data are not used for this task since no non-cancer samples are available. Table 2 gives the characteristics of this dataset. This dataset is divided into a training set of 5000 samples and a test set of 1450 samples for the binary classification task and 980 samples for the multi-class classification task by preserving the proportion of cancer/non-cancer samples and the type of cancer.

**Table 2:** Characteristics of the TCGA dataset

Disease	Size	Cancer	Non-cancer	Prior
BRCA	1214	1101	113	0.91
KIRC	610	538	72	0.88
LUAD	592	533	59	0.90
UCEC	574	551	23	0.96
THCA	560	502	58	0.89
LUSC	551	502	49	0.91
PRAD	550	498	52	0.90
HNSC	544	500	44	0.92
LGG	510	510	0	1
OV	374	374	0	1
LIHC	371	371	0	1
Total	6450	5980	470	0.927

The columns represent respectively the type of tissues (Disease), the numbers of samples (Size), cancer samples (Cancer), non-cancer samples (Non-cancer) and the proportion of the majority class (Prior). This dataset contains only patient data

### Sensitivity analysis

The NN is one of the most complex models to optimize in machine learning because of the high number of hyper-parameters to tune. We investigate the impact of the main hyper-parameters on the model accuracy for the classification tasks previously defined (these hyper-parameters are described in Sect. 5.1). Hyper-parameters defining the architecture of the NN (number of layers, number of neurons, batch normalization, dropout) and controlling the training (optimizer, learning rate, L1 or L2 regularization, batch size) are tested by a random search procedure. A range of tested values for each hyper-parameter is defined and reported in Table 3. At each iteration, for each hyper-parameter, a value is randomly drawn from its range following a uniform distribution. A NN is constructed and trained with these parameters from 80% of the training set, and its accuracy is estimated on the remaining 20%. This procedure is iterated more than 10,000 times for each classification task.

**Table 3:** Tested values of the hyper-parameters and their best values for the pan-cancer prediction tasks

Hyper-parameters	Tested range	Microarray	TCGA cancer pred.	TCGA type pred.
Nb layers	[1, 20]	4	5	4
Nb neurons	[20, 2000]	600-600-600-60	500-500-500-500-50	700-700-700-50
Batch norm.	Yes/no	No	Yes	Yes
Dropout	[0, 0.8]	0	0	0
Optimizer	SGD/RMSprop/ADAM	SGD	SGD	SGD
Learning rate	$[1^{-6},1]$	$10^{-3}$	$10^{-3}$	$5.10^{-2}$
L1 regularization	$[0,1^{-2}]$	0	0	0
L2 regularization	$[0,1^{-2}]$	0	0	0
Batch size	[8, 1024]	8	32	8

**Fig. 2:**
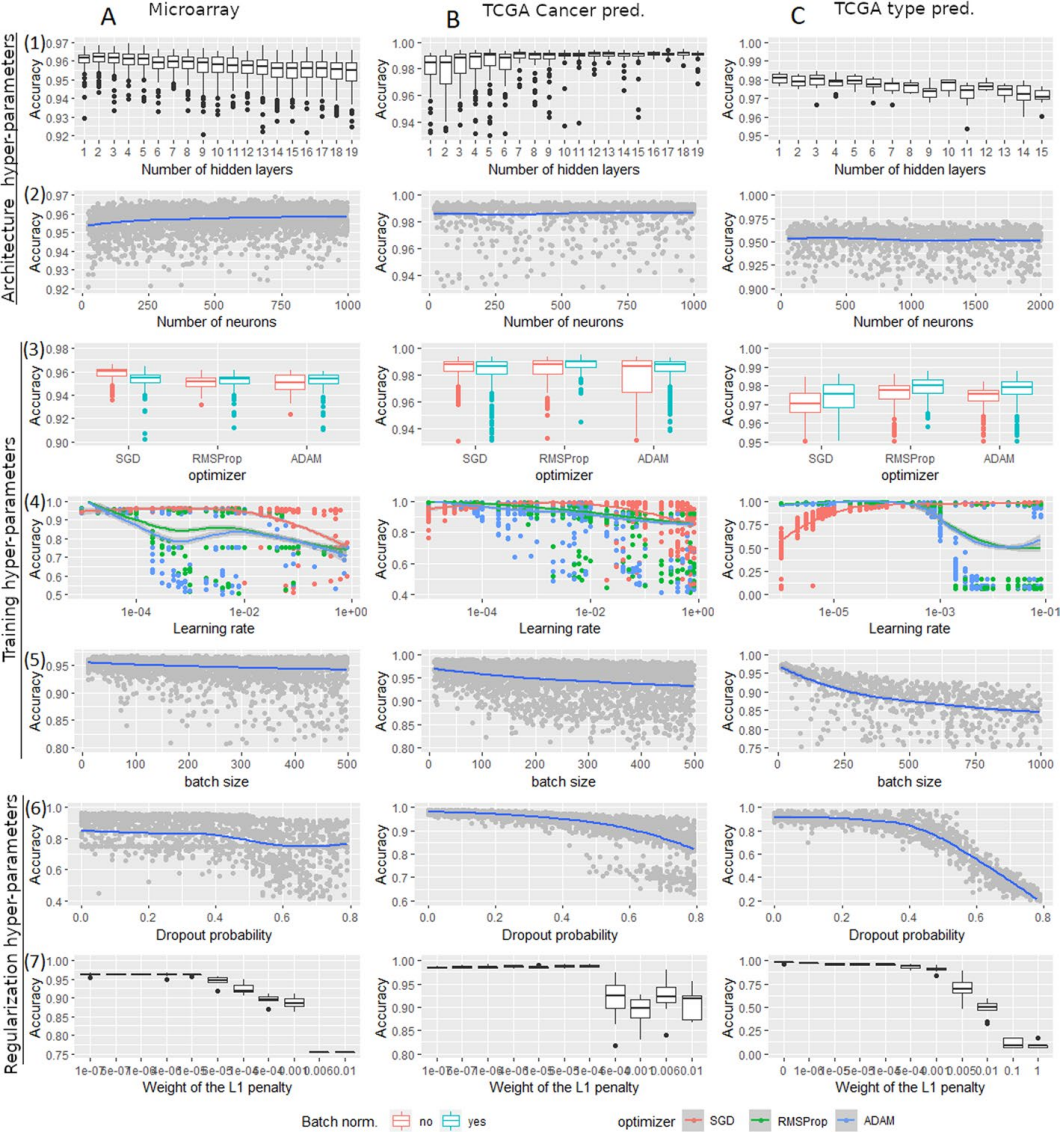
Analysis of the impact of each hyper-parameter on the accuracy of the model

The results of these experiments are represented in Fig. 2. Each row (1–7) of this figure represents the impact of a hyper-parameter on the model accuracy, and each column (A–C) represents the results on a classification task. A boxplot represents the performances obtained for each tested value for the number of hidden layers, optimizer, and weight of the L1 penalty. For the number of neurons, batch size and dropout probability (rows 2, 5 and 6), each point represents a DL model (represented by its hyper-parameter value and accuracy). The blue curve is the loss interpolation that gives the tendency of the accuracy. The learning rate figures (row 4) use the same representation; the three tested optimizers are differentiated using distinct colors.

The results from the two first rows show that the architecture of the network has few impacts on its performance. There is less than 2% difference between the best and the worst boxplot in the number of hidden layers figures (row 1) and less than 1% in the evolution of the loss curve in the number of neurons figures (row 2). The best accuracy for microarray (column A) and TCGA type prediction (column C) tasks is reached by small networks. Beyond five hidden layers, the accuracy is decreasing. It is well-known that deep networks may be difficult to train correctly because of the gradient vanishing problem. The standard method to avoid this problem consists in using residual connections to preserve a good gradient retro-propagation. We tested this approach on networks from 10 to 20 hidden layers and noted that the residual connections do not improve the accuracy. For the TCGA cancer prediction task (column B), the accuracy slightly increases from one layer to four layers and becomes stable for deeper networks.

The results from rows 3–5 show that the gradient descent greatly impacts the performances. Three standard optimizers are tested and compared: the stochastic gradient descent with momentum (SGD), RMSprop, which uses the squared gradients to scale the learning rate, and Adam, which combines the principles of momentum and RMSprop. The three optimizers may produce the best accuracy if they are well-tuned. On the learning rate figures (row 4), we see that the behaviors of RMSProp and ADAM are very similar. Both need a much smaller learning rate value than SGD, and models may reach good or very poor accuracy for some ranges of the learning rate. Note the binary aspect of this observation: either the gradient descent has a correct trajectory, and we obtain good performances, either the optimizer fails, and the performances are awful. SGD is much more stable and never produces very bad results except for extreme values of learning rate. The optimizer boxplot figures (row 3) cannot be used to compare the performances of the optimizers since they represent the accuracies obtained with all tested learning rates. We should keep only the accuracies with the correct learning rate range for each optimizer for a fair comparison. These figures are useful for comparing networks’ accuracy with and without batch normalization. We see that batch normalization has a small impact on the performances. It is beneficial only for the TCGA type prediction task (column C). The batch size has a significant impact on the performances. It appears that it is negatively correlated with the accuracy, especially on the TCGA type pred task. Small batch size produces better results for all classification tasks.

The results from the two last rows show the impact of the regularization methods on the performances. The dropout and L1 penalty (rows 6 and 7) does not change the accuracy for small values and decrease the accuracy for large values. Note that the results of the L2 penalization (not shown in the figure) are very similar to those of the L1 penalization. In all of our experiments, we do not identify cases where the regularization methods improve the performances.

### Comparison of the deep learning approach with the state-of-the-art

The performance of neural networks with the optimal hyper-parameters selected in the previous (see Table 3) is evaluated on the three classification tasks and compared with the state-of-the-art of machine learning: extreme gradient boosting (XGBoost), least absolute shrinkage and selection operator (LASSO), random forest (RF), and support vector machine (SVM) with linear (SVMlin) or Gaussian (SVMrad) kernel. A t-test based selection of the most discriminative gene has been used for the classical machine learning method. The number of selected genes and the hyper-parameters of these methods have been tuned in an internal tenfold cross-validation loop. Table 4 gives the accuracies obtained by NN and classical machine learning methods on the three pan-cancer classification tasks. A paired t-test tests the significance of these results. NN has the best accuracy on the three tasks. We note that on the TCGA dataset, the difference of accuracy between NN and others methods is non-significant for the cancer prediction task and small for the type prediction task. All classifiers obtain the same level of accuracy, except LASSO. Note that the TCGA classification tasks are easier to achieve when the size of the training set is 5000, the classifiers reach 99% of accuracy. There is no space for significant improvements. Concerning the microarray dataset, NN is significantly better than other classifiers (*p* value < 0.001) with a large margin (from 94.75 for SVMrad to 96.18 for NN).

**Table 4:** Accuracies obtained by NN and classical machine learning methods on the three classification tasks

Classifier	Microarray	TCGA can.	TCGA type
XGBOOST	92.56 ± 0.29	99.03 ± 0.27	98.50 ± 0.14
LASSO	93.79 ± 0.35	97.70 ± 0.22	98,46 ± 0.14
RF	93.76 ± 0.29	98.41 ± 0.11	97,39 ± 0.21
SVMlin	93.81 ± 0.19	98.45 ± 0.09	98,70 ± 0.09
SVMrad	94.75 ± 0.25	98.66 ± 0.11	98,51 ± 0.09
NN	96.18** ± 0.18	**99.09** ± 0.25	98.89** ± 0.18

Bold highlights the methods with the best accuracy

The symbol ** indicates that the accuracy of NN is significantly higher than the other methods (*p* value $<0.01$ from the paired t-test)

**Fig. 3:**
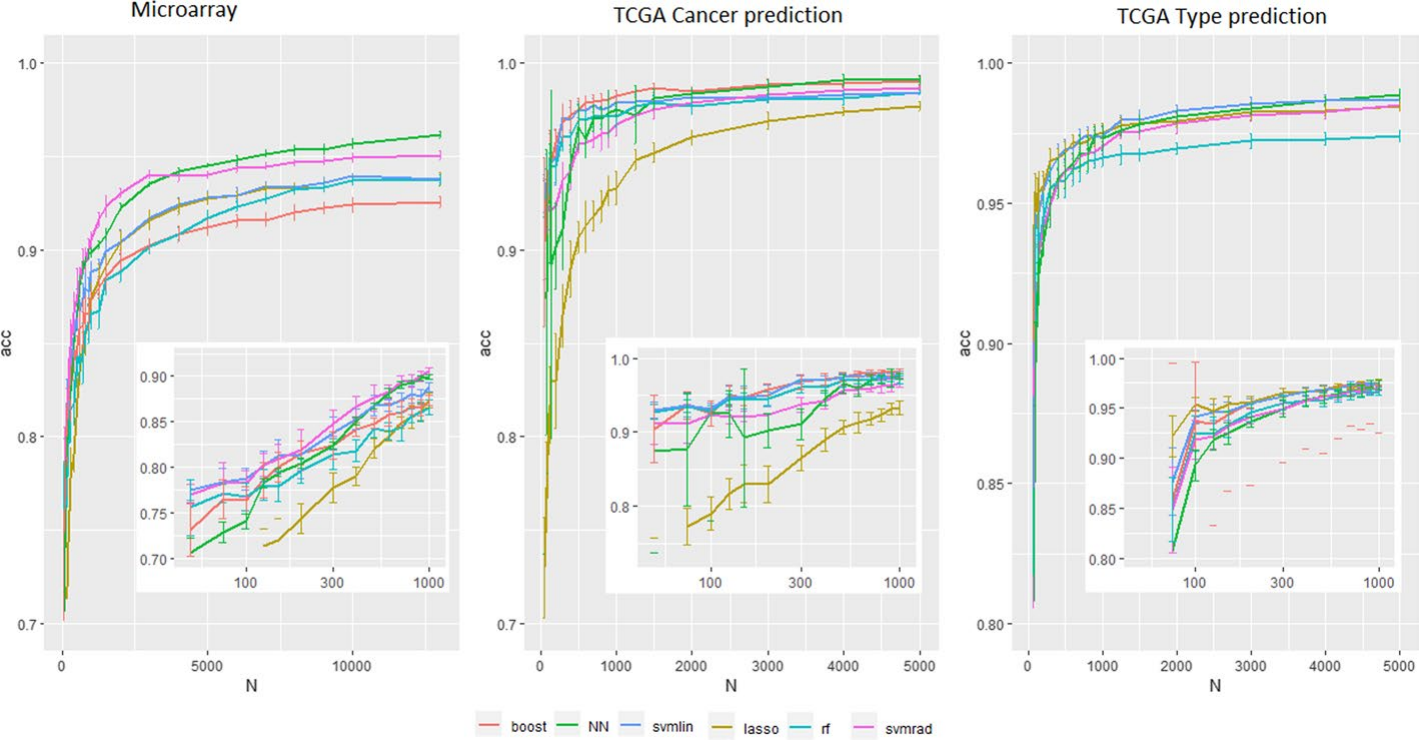
Accuracy of state-of-the-art ML and NN models in function of the training set size

The performance of the models is highly dependent on the number *N* of available examples for the training. Figure 3 shows the accuracy of NN, XGBoost, LASSO, RF, and SVM as functions of the size of the training set on both datasets. We clearly see that NN gives worse results for very small datasets ($N<300$) than the state-of-the-art. From $N=300$ to $N<3000$, NN obtains performances equivalent to the other machine learning methods. From $N>3000$, NN becomes the best method, and the difference with the other methods increases with increasing *N*. On the microarray dataset, from $N>5000$, the gain of performance of classical machine learning methods coming from the increase of the training set size is small, whereas the accuracy gain of NN is much larger. NN takes advantage much more from very large training sets.

NN has similar results for specific cancer classification to other methods (detailed results in supplementary materials). None of the tested methods is statistically better than the others. The size of the training sets is between 500 and 1000; these results are therefore coherent with the curves in Fig. 3 where all methods give similar results in the area defined by ‘$500< N < 1000$’.

### Supervised transfer learning

We investigate the transfer learning approach to improve the performances of the NN [15, 22]. There are two types of data in the context of transfer learning: the source and the target data. The target dataset represents the data of interest on which a classification task is defined. The idea of transfer learning is to transfer relevant information from the source model to the target model to make it more efficient. The classification task associated with the source data must be identical or related to the target task.

#### Between cell lines and patient data

This subsection evaluates the benefit of supervised transfer learning between cell lines and patient data on the microarray dataset. Although we mixed these two types of data to learn an accurate model for cancer prediction in the previous section, it clearly appears that cell line data and patient data do not follow the same distribution. We highlight this point in the following experiments. A NN is only trained on the training cell line data and tested separately on the cell line and patient test data. The same experiment is done with a NN trained only from training patient data. All the procedure is iterated 10 times, this means that 10 NN are trained on cell line data and tested on patient data for each value of *N* and *F* The results presented in the Table 5 show that cell line data are easier to predict than patient data. Models reach almost 100% of accuracy for cell line data prediction with $N=4527$ whereas models are still below 95% for patient data prediction with more than twice training examples. It is not so surprising, since we know that the biology in real persons is much more variable and complex than in cell lines, the classification task is therefore harder. The second and most important result is that a model trained on cell line (resp. patient) data cannot be applied on patient (resp. cell line) data; the accuracy falls from 99% (resp. 94%) to 66% (resp. 59%).

**Table 5:** Accuracy of model trained on patient or cell line data

	Test	
	Cell line	Patient
Training		
Cell line	99.36 ± 0.12	66.33 ± 3.17
Patient	59.29 ± 7.25	94.80 ± 0.42

Although the distribution of cell lines and patient data is different, the tissues’ biology and gene expression patterns should be similar. We, therefore, hypothesize that a transfer of information would be possible between cell line models and patient models. Indeed, gene interactions and expression signatures should be related between cell lines and patient data and transferable from one type of data to another. We test this hypothesis in the following experiment. The cell line data are considered as the source and the patient data as the target. The source and target classification task are the same: the prediction of the presence of cancer. A model is trained using all training cell line data; its accuracy must approach the accuracy reported in the Table 5. Then, the first *F* layers are frozen ($F \in \{0,\dots ,4 \}$), i.e., the weights of these layers become fixed. Finally, a second training of the network, called fine-tuning, is performed using a subset of *n* training patient data ($n \in [25,5000]$). Note that the unfrozen layers are not reinitialized during the fine-tuning procedure, and the weights from the first training are kept. Ten fine-tuned models are trained from this procedure for each value of *F* and *n*, and ten baseline models are trained for performance comparison. The baseline models correspond to NN trained directly from the *n* training patient data used for fine-tuning. The same experiment is also done with the patient data as the source and cell line data as the target for testing the transfer from patient model to cell line model.

**Fig. 4:**
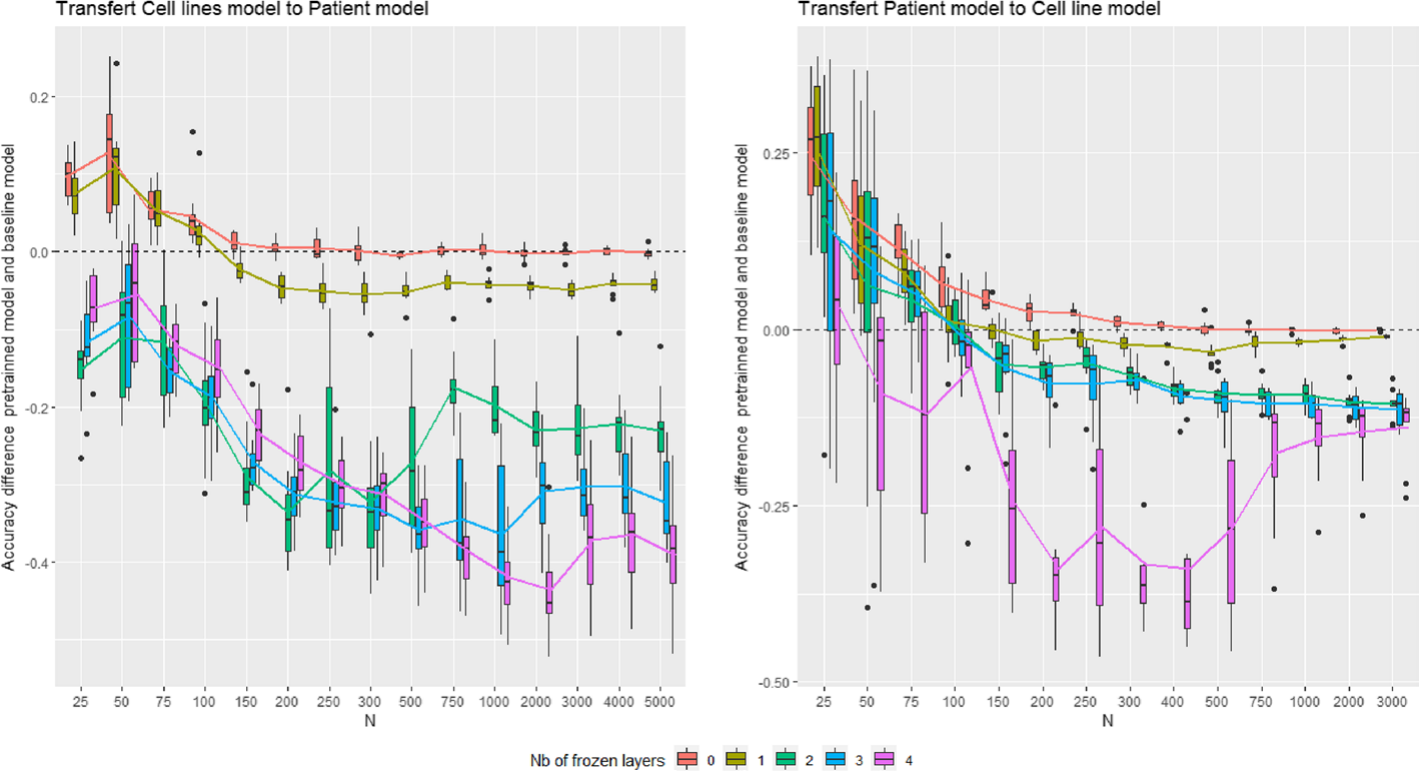
Transfer between cell lines and patient data. Accuracy difference between pre-trained model and baseline model. The colors represent the number of frozen layers in the pre-trainned model

Figure 4 gives the results of both transfer learning experiments. The curves represent the gain of accuracy provided by transfer learning, i.e., the accuracy of fine-tuned models minus the accuracy of baseline models. Each curve represents the transfer learning performance with different values of *F*. Each boxplot of the Fig. 4 plot represents the accuracy gains of 10 fine-tuned NN. We show that the best performances are obtained for $F=0$, corresponding to the special case of transfer learning called pre-training. For $F=1$, the curve is just below the pre-training curve, the other curves ($F=2,3,4$) are located much lower (particularly from cell line model to patient model). The more layers are frozen, the worse the performances of transfer learning are. All curves, except the pre-training curve, show a phenomenon of negative transfer learning for some values of *n*. Transfer learning decreases the performance of the models instead of increasing it. This phenomenon is bigger with many frozen layers. If the pre-trained layers are not relevant for the target data, it will be difficult to correct the weights of the NN during the fine-tuning step if many layers are frozen. The benefit of transfer learning highly depends on the size of the training set *n* and is particularly interesting for small training sets. With $n=25$ and $F=0$, for the transfer from cell line to patient model, the baseline model obtains an accuracy of 64.54%, and the fine-tuned model reaches 74.18%; for the transfer from patient to cell line model, the baseline model obtains an accuracy of 55.58%, and the fine-tuned model reaches 80.74%. The baseline models learned nothing with a very small dataset; their accuracy is around the proportion of the majority class, whereas fined-tuned models provide more relevant predictions. Pre-training is beneficial for $n<200$ for cell lines to patient transfer and $n<400$ for patients to cell line transfer. After these thresholds, pre-trained models obtain the same accuracy as the baseline. However, we note that the convergence of the gradient descent during fine-tuning takes fewer epochs than in the baseline models training. The pre-training can be interpreted as an initialization of the NN to a point in the parameter space where the gradient descent will converge faster and more efficiently in a small training set scenario. We also notice that the transfer from patient to cell lines is more efficient than from cell lines to patient; the accuracy gain is up to 0.25 in the first situation and 0.15 in the second one.

#### Between different types of cancer

In this section, we test the transfer learning between different types of cancer. Unlike the transfer between cell lines and patient data cases, the gene expression profiles of different types of cancer can be different. It could be hard to find relevant expression patterns to transfer from one cancer to another. However, some studies on pan-cancer data point out that it is possible to identify a global signature of cancer [20]. In the next two sets of experiments, we evaluate the performance of transfer learning, firstly from all types of cancer to a specific one and secondly from one type of cancer to another one. The previous experiments show that supervised transfer learning is better when no layers are frozen, so only supervised pre-training is considered in the following experiments. Since the target data is limited to a specific type of tissue, the target dataset is small (from 371 to 4283). All accuracies are therefore estimated by stratified tenfold cross-validation.

*From pan-cancer to specific cancer*

**Fig. 5:**
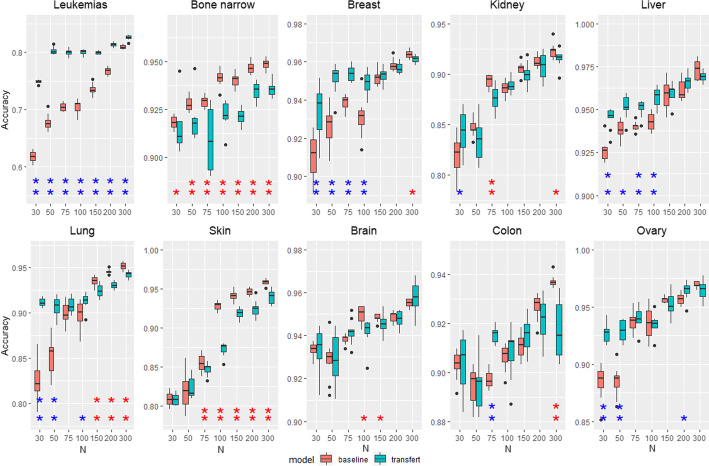
Transfer from pan-cancer to a specific cancer for microarray data. Accuracy of pre-trained model (red) and baseline (blue). The single (resp. double) star indicates that the accuracy difference is significant with a *p* value of 0.05 (resp. 0.01)

**Fig. 6:**
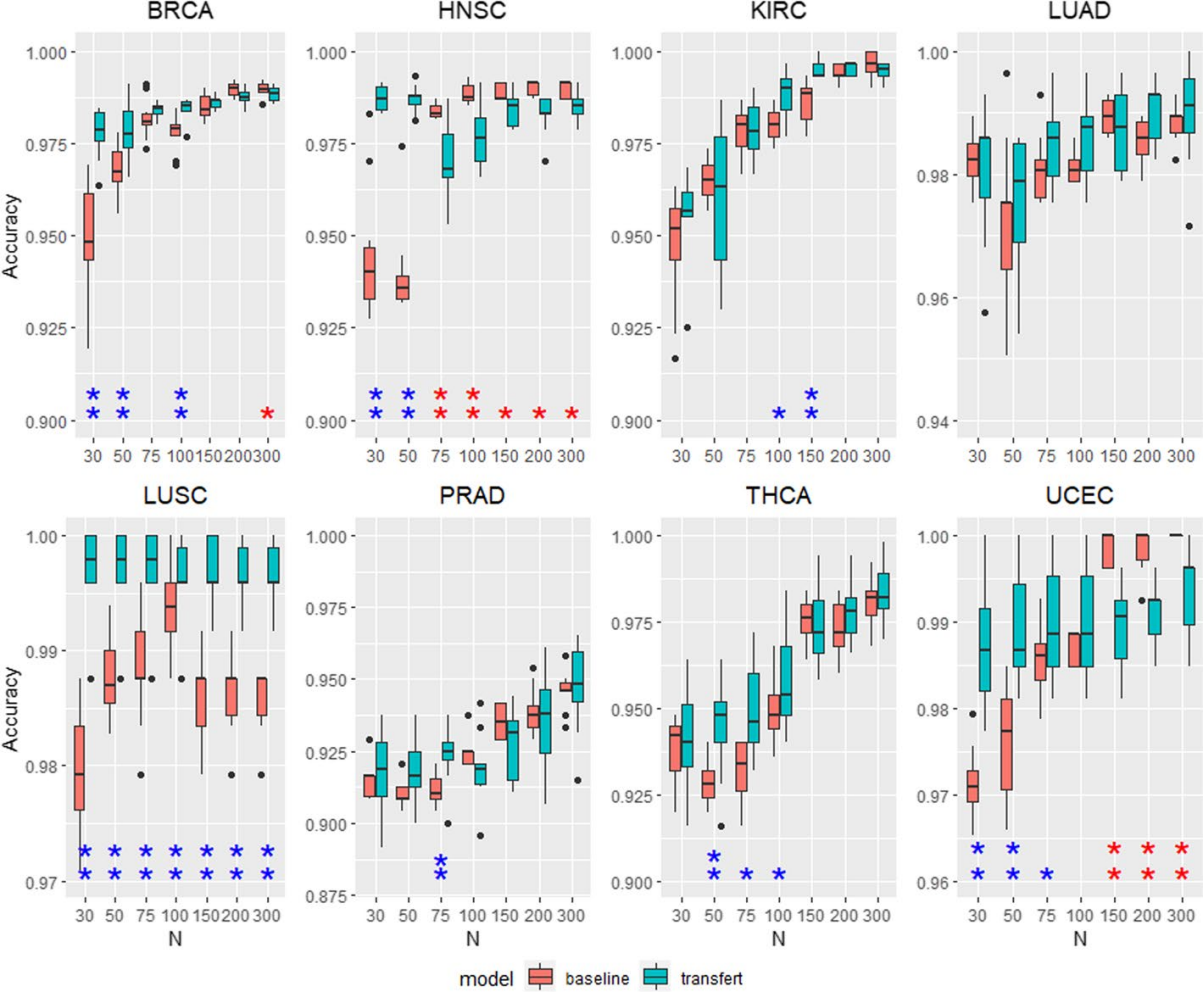
Accuracy of pre-trained model (red) and baseline (blue) for TCGA data. The single (resp. double) star indicates that the accuracy difference is significant with a *p* value of 0.05 (resp. 0.01)

This experiment evaluates the transfer from a model trained on all types of cancer to a specific one. For a given target type of cancer, all training examples except the examples of the target type are used to train a source model. Then, the source model is transferred to the target model that is fine-tuned with a subset of *n* training examples from the target data. The accuracy of the target model to detect cancer in the target data is evaluated and compared with the baseline model trained directly using the *n* training examples of the target tissues. Figures 6 and 5 show respectively the results on the TCGA and microarray datasets. The accuracy of baseline and fine-tuned models is represented respectively in red and blue as a function of *n*. For each value of *n*, the significance of the difference of accuracy between baseline and fine-tuned models is estimated by a paired t-test. The one star (resp. double stars) symbol means that the difference is significant with a *p* value of 0.05 (resp. 0.01) and the color of the star indicates the best model (red for baseline, blue for fine-tuned).

Transfer learning performance is not as good as in the cell lines—patients case. The effectiveness of transfer learning depends on the type of cancer. We identify three types of results. The first one concerns cases where transfer learning is beneficial, the accuracy of fine-tuned models is higher than baselines for small *n*. It gathers leukemias, breast, liver, lung, ovary for microarray data and BRCA, HNSC, LUSC, THCA, UCEC for TCGA data. As in the previous experiment, the less data there is, the more useful transfer learning is. In the second type of results, transfer learning does not change the performances. The accuracy of fined-tuned and baseline models is equivalent. It concerns kidney, brain, colon for microarray data and KIRC, LUAD, PRAD for TCGA data. The last type presents negative transfer; the fine-tuned models have lower accuracy than baselines. It concerns only bone marrow and skin for microarray data. Note the case of the lung cancer in microarray data and HNSC, UCEC for TCGA data, where transfer learning is clearly beneficial for small *N*, but becomes negative for larger *N*. We point out that the negative transfer is very small in these special cases. The accuracy difference is less than 1% for HNSC and UCEC, and less than 2% for lung, which is much lower than the gain of accuracy for small *N*.

*From one cancer to another*

**Fig. 7:**
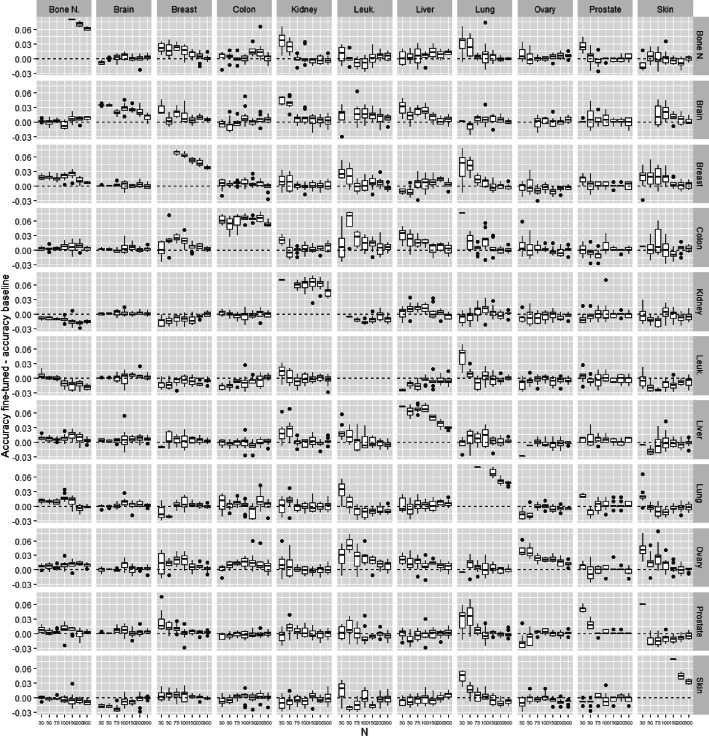
Transfer between two types of cancer for microarray data. Accuracy difference between pre-trained model and baseline model in function on the training set size

**Fig. 8:**
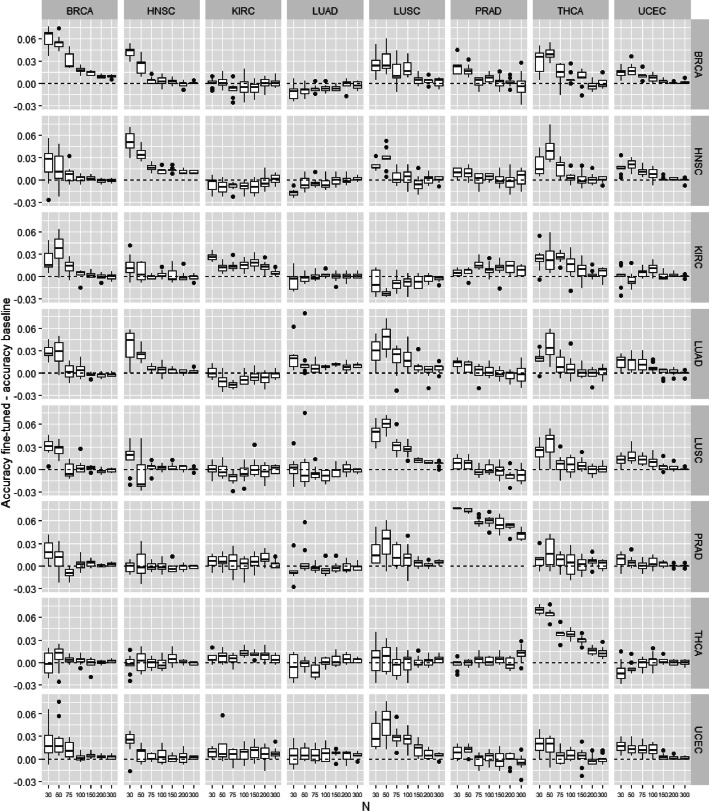
Transfer between two types of cancer for TCGA data. Accuracy difference between pre-trained model and baseline model in function on the training set size

In this experiment, we test the possibility of transfer learning from specific cancer to another one. All training examples from the source data are used to train the source model. Then, this source model is transferred to the target model that is fine-tuned with a subset of *n* training examples from the target data. The accuracy of the target model to predict the presence of cancer in the tissue of the target type is evaluated and compared with the baseline model trained directly from the *n* training examples of the target tissue. Figures 7 and 8 show the difference of accuracy between the baseline and fine-tuned model for all pairs of source/target combination on the TCGA and microarray datasets. The diagonal represents results where source and target are the same. This case is equivalent to train a model from a training set containing the source and target set. It is not surprising that the fine-tuned models obtain much better accuracy than the baseline. In the majority of the other cases (source different from target), the transfer learning does not improve the baseline. There is also almost no negative transfer. However, we note some cases where fine-tuned models are significantly better than baselines: “brain to kidney”, “bone marrow/breast/skin to lung”, “ovary to skin” in microarray data, and “UCEC to THCA / LUSC” in TCGA data. It would be interesting in future experiments to confirm these results and check whether these special cases are just some artifacts in the set of experiments or relevant biological information can really be transferred in these specific cancers.

### Unsupervised transfer learning

**Fig. 9:**
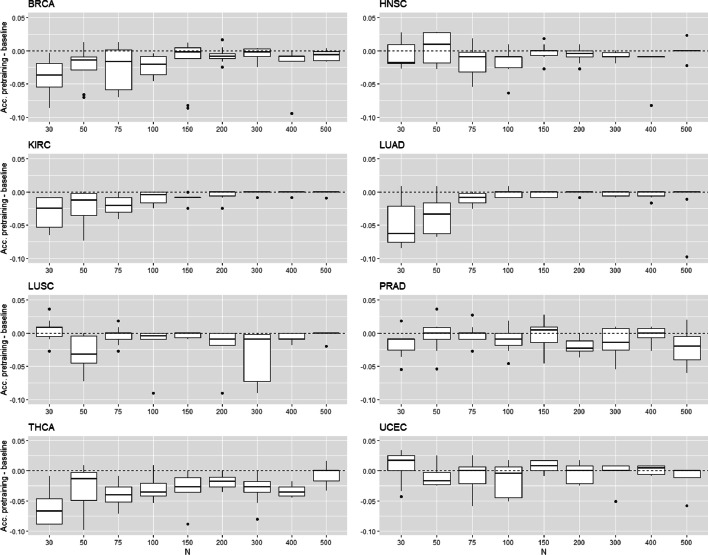
Unsupervised transfer for TCGA data. Accuracy difference between pre-trained model and baseline model in function on the training set size

**Fig. 10:**
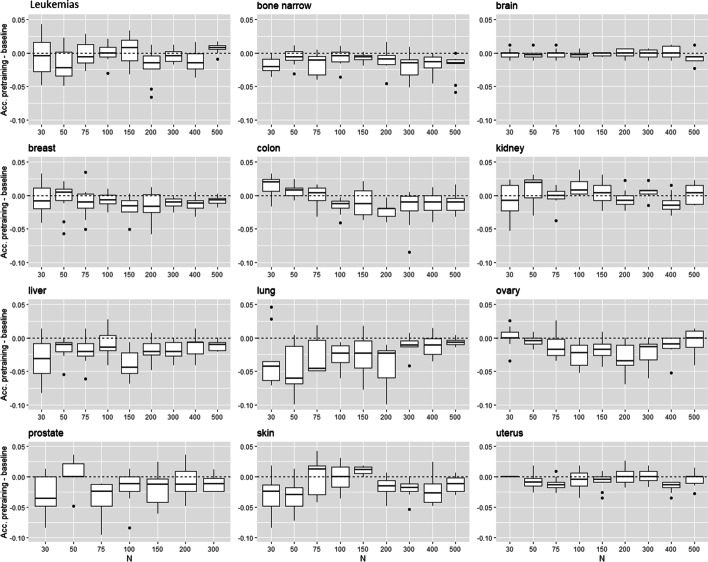
Unsupervised transfer for microarray data. Accuracy difference between pre-trained model and baseline model in function on the training set size

This section evaluates the performance of unsupervised transfer learning on cancer prediction tasks. We consider a small target labeled dataset ($N \le 500$) and a large source of unlabeled dataset ($N'=10000$ for microarray and $N'=3000$ for TCGA). Each layer of the NN is successively trained through a denoising autoencoder (DAE) with the source dataset. Then, the model is fine-tuned with a subset of *N* target examples. The accuracy of this model to predict the presence of cancer is evaluated and compared with the baseline model trained directly from the *N* target examples. Figures 9 and 10 show the difference of accuracy between the baseline and fine-tuned model for each type of tissue. In these figures, we show only the performance of the pre-training procedure, i.e., no layer has been frozen during the fine-tuning step. For all types of tissue, the accuracy of fine-tuned models is never higher than the baselines. We identify two types of results. In the first one, the accuracy of fine-tuned and baseline model is similar whatever the number of training labeled examples (HNSC, LUSC, PRAD, UCEC for TCGA and leukemias, bone marrow, brain, breast, colon, kidney, ovary, uterus for microarray). The pre-training has no impact on the model training. We reach the same performance as the baseline model with random initialization of the hidden layers. In the second type of results, the performance of fine-tuned models is worse than the baselines for very small *N* and increases with *N* to reach the performance of the baseline (BRCA, KIRC, LUAD, THCA for TCGA and liver, lung, prostate, skin for microarray). The pre-training has a negative transfer effect and damages the model’s training. A minimum number of target examples is needed to put back the gradient descent on the right way and reach the performances of the baseline. We also tested the unsupervised transfer learning by freezing some hidden layers during the fine-tuning step. In this setting, the negative transfer effect cannot be fixed, and the accuracy of the fined-tuned models is therefore much lower than the baseline models. In conclusion, we identify no advantage of unsupervised transfer learning in our experiments.

## Discussion

The training of a NN is more complex than with the other machine learning models because of the large number of hyper-parameters to optimize. Although the optimal value of the hyper-parameters depends on the data, the results of our extensive set of experiments lead to some general recommendations. We recommend using a small batch size and SGD as the optimizer, which is more stable than ADAM or RMSprop. Since the architecture of the network has few impacts on the performance, it seems not necessary to spend many resources to optimize it. Note, this flexibility can be exploited to make the NN interpretable by constraining its architecture with biological knowledge like in [10]. Surprisingly, the regularization methods do not reduce overfitting. We recommend focusing on the optimization of the learning rate and batch normalization.

The comparison with the state-of-the-art shows that a well-tuned NN is competitive with the other machine learning methods if the training set contains hundreds of examples ($N>300$ in our experiments). For small datasets, classical methods are more accurate. The NN shows its advantages only for very large datasets ($N>3000$). Since most of the current gene expression datasets are far from this size, the usefulness of NN in this context can be discussed. However, the genomics data are considered as a pillar of the future precision medicine [23] and the production of data is strongly increasing over the world. In the next ten years, the size of the gene expression datasets will gain one or more orders of magnitude, potentially making NN the method of reference. Note that this is what happened in image analysis and natural language processing, where with the increasing of the training sets size, the NN over-performed the other methods and became the state-of-the-art. We may expect the same phenomenon in genomics data analysis.

The results on transfer learning are particularly interesting and promising. We show that it is possible to transfer a cancer signature from one condition to another. The most efficient transfer is between cell lines and patient data. Even if the distribution of cell lines and patient data is different, the signature of the presence of cancer is related and can be transferred between these two conditions. All successful transfers use a pre-training approach, i.e., all layers must be fine-tuned. This means that a signature identified in one condition cannot be directly used in another one; the model must be adjusted on the distribution of the target data through fine-tuning. We also note that the transfer from patient to cell lines is more efficient than from cell lines to patient. An explanation could be that since the patient data are more complex and diverse than cell lines, the NN trained from cell lines cannot capture all relevant expression patterns and make the generalization to patient data more complex. Since the production of cell line data is much easier than patient data, transfer learning may be a promising approach to develop quickly reliable predictive models. For a given disease, we could produce a large number of cell line data to construct a NN, and then the model would be fine-tuned on a smaller patient dataset to obtain an accurate predictive model of the disease.

The transfer between a pan-cancer model to a specific cancer model is also possible, even if there are no samples of specific cancer in the pan-cancer data. This means that the NN can identify a general signature of the presence of cancer from pan-cancer data. It is important not to over-interpret this result. We do not claim that the NN finds a common biological signature shared by all cancers. The different types of cancer are very heterogeneous and may be biologically very different. We claim that the NN finds a common informative signature of cancer in the gene expression data. That is different from a common biological signature since the NN (or all other machine learning models) identifies only correlations between gene and output and no causalities. However, this informative signature can be transferred to produce an accurate classifier with a very small dataset. This transfer is less robust than in the cell line/patient conditions. The transfer does not improve performance for some cancers and can even produce a negative effect. For the moment, we do not identify the conditions that make the transfer successful or not; this point will be investigated in future works. Even if we cannot ensure the transfer efficiency for all tissues, it should be possible to get rid of the negative effect with domain adaptation methods [24]. The transfer from pan-cancer data may particularly help for rare cancers. Even with the rise of the capacity of genomics data production, the datasets of rare cancers will still be small. With this approach, we could transfer signatures from pan-cancer data to small rare cancer data.

The transfer from a given cancer to another one does not work. We assume that the NN identifies a signature that is too specific to the source cancer. Since the biology between two cancers may be very different, the signature cannot be transferred to another one. In pan-cancer data, the NN analyses a wide variety of cancer and may identify a general signature. That is not the case here.

Our results show that unsupervised transfer learning does not improve the performances of the baseline. This conclusion is in opposition with previous studies that claim that the embeddings learned by autoencoder from unlabeled data improve the accuracy of the NN after fine-tuning [25]. We can explain this difference because only the procedure, including unsupervised pre-training and fine-tuning, is tested in most articles. There is no comparison to a model learned without pre-training. We assume that dropping the pre-training would not affect the model’s accuracy. Another explanation is that a sub-optimal model may benefit from unsupervised pre-training. We observe in our experiments that models trained with no optimal hyper-parameters may improve their accuracy with unsupervised pre-training. If we use the optimal hyper-parameters, the unsupervised pre-training may not ensure the increasing of the accuracy of the baseline. The conclusion is that if we use a well-optimized NN, there is no benefit to unsupervised transfer learning.

## Conclusion

In this study, we evaluated the performance of the deep learning approach for cancer prediction from gene expression data based on an exhaustive set of experiments. We provided several recommendations to optimize the NN construction and training. Given the size of the current gene expression datasets, NN is competitive with other machine learning methods but not significantly better. However, with the increase in the size of the datasets, NN will likely become a reference method in the next years.

We showed that transfer learning is possible for gene expression data, mainly between cell lines and patient data, and from pan-cancer to specific cancer. The approach is very promising to develop accurate models, especially for rare cancers where large datasets will never be available. It is important to perform complementary experiments in order to confirm these results and identify efficient transfer conditions. In this paper, we focus on transfer learning based on pre-training and fine-tuning; however, it could also be interesting to investigate more sophisticated transfer learning methods. We could use the domain adaptation approach that aligns the distribution of the source and target data in the hidden layers to make the transfer more efficient [24]. The integration of domain knowledge could also be a solution to control and focus the transfer on the most critical information for the prediction task.

## Methods

### Deep neural network

Given a classification task with *K* classes, a classifier is a function that associates a class to an input vector: ${\mathcal {F}}:x \mapsto y$. In our work, $x\in {\mathbf {R}}^{p}$ is a gene expression profile, $y \in \{c_1, \ldots ,c_K\}$ is the predicted class corresponding to the phenotype, and ${\mathcal {F}}$ is a deep neural network. In the context of gene expression data, we use a MLP architecture with *L* layers. In this architecture, the neurons are organized in layers, where each neuron is connected to all neurons of the previous layer and all neurons of the next layer. The input layer receives a gene expression profile, each neuron takes the expression of one gene. The output layer returns the probabilities to belong to each class (one neuron for each class). The activation of the i-th neuron of the layer *l* can be expressed as: $a_i^{(l)} = g \left( \sum _{j=1}^{n_{l-1}} a_j^{(l-1)} w_{ji}^{(l)} + b_i^{(l)} \right)$, where $w_{ji}^{(l)}$ is the weight of the connection from the j-th neuron of the layer ($l-1$) to the i-th neuron of the layer *l*, $b_i^{(l)}$ is the bias of the i-th neuron of the layer *l* and $n_l$ the number of neurons in the layer *l*. We denote $z_i^{(l)} = \sum _{j=1}^{n_{l-1}} a_j^{(l-1)} w_{ji}^{(l)} + b_i^{(l)}$ the input of the i-th neuron of the layer *l*. The activation function, *g*, in this work, corresponds to the rectified linear unit function (ReLU) $g(z)=max\{0,z\}$ for the hidden layers and the softmax $g(z^{(L)})_k=exp(z_k^{(L)})/\sum _{j=1}^{K} exp(z_j^{(L)})$, where $z^{(L)} = \{z_1^{(L)},\ldots , z_K^{(L)}\}$, for the output layer. The expression profile representing a patient at the input of the network is noted $a^{(0)}$, the posterior probability of each class $c_k$ estimated by the network is noted as $a_k^{(L)}=g(z^{(L)})_k$ and the prediction of the neural network is ${\mathcal {F}}(x)=argmax_k\{a_k^{(L)}\}$.

The loss function to minimize during the training is the weighted cross-entropy defined by $Loss = -\sum _{i=0}^{N} \sum _{k=0}^{K} \alpha _k I(y_i=k) log( a_{k,i}^{(L)})$ where *I* is the indicator function returning 1 when $y_i=k$ and 0 otherwise, $\alpha _k$ is the weight of each class that is inversely proportional to the proportion of the class in the training set. It is common to add dropout or L1/L2 penalty to reduce the overfitting of NN. Dropout consists of switching off a random subset of the inputs or hidden neurons, i.e. set their output to 0; the proportion of neurons is a hyper-parameter to set [26]. The L1/L2 regularization consists of adding a penalty that corresponds to the L1 or L2 norm of the weights of the NN. This penalty is controlled by a hyper-parameter to set. Another simple method to reduce overfitting is the use of early stopping. The loss function on a validation set is monitored at each epoch; the gradient descent stops when the validation loss increases.

### Transfer learning

Transfer learning aims to deal with the problem of small size training datasets. It consists of transferring information from the source domain to the target domain in order to perform a target task [22].

A domain $D = \{ X, P(X)\}$ is defined by the feature space *X* and a probability distribution associated to this space *P*(*X*). A task ${\mathcal {T}} = \{ Y, {\mathcal {F}}(X)\}$ is composed of two parts: the label space *Y* and the target prediction function ${\mathcal {F}}(X)$. ${\mathcal {F}}(X)$ can be considered as a conditional probability function *P*(*Y*|*X*). Given a target learning task ${\mathcal {T}}_t = \{ Y_t, {\mathcal {F}}_t(X)\}$ based on a target domain $D_t = \{ X_t, P_t(X)\}$ and a source task ${\mathcal {T}}_s = \{ Y_s, {\mathcal {F}}_s(X)\}$ based on a source domain $D_t = \{ X_s, P_s(X)\}$, the transfer leaning goal is to improve the performance of the model that learns the task ${\mathcal {T}}_t$ in $D_t$ using the knowledge in $D_s$ and ${\mathcal {T}}_s$. Based on the above definition of the transfer learning, a small number of labeled data in the target domain is required to induce the target predictive function.

**Fig. 11:**
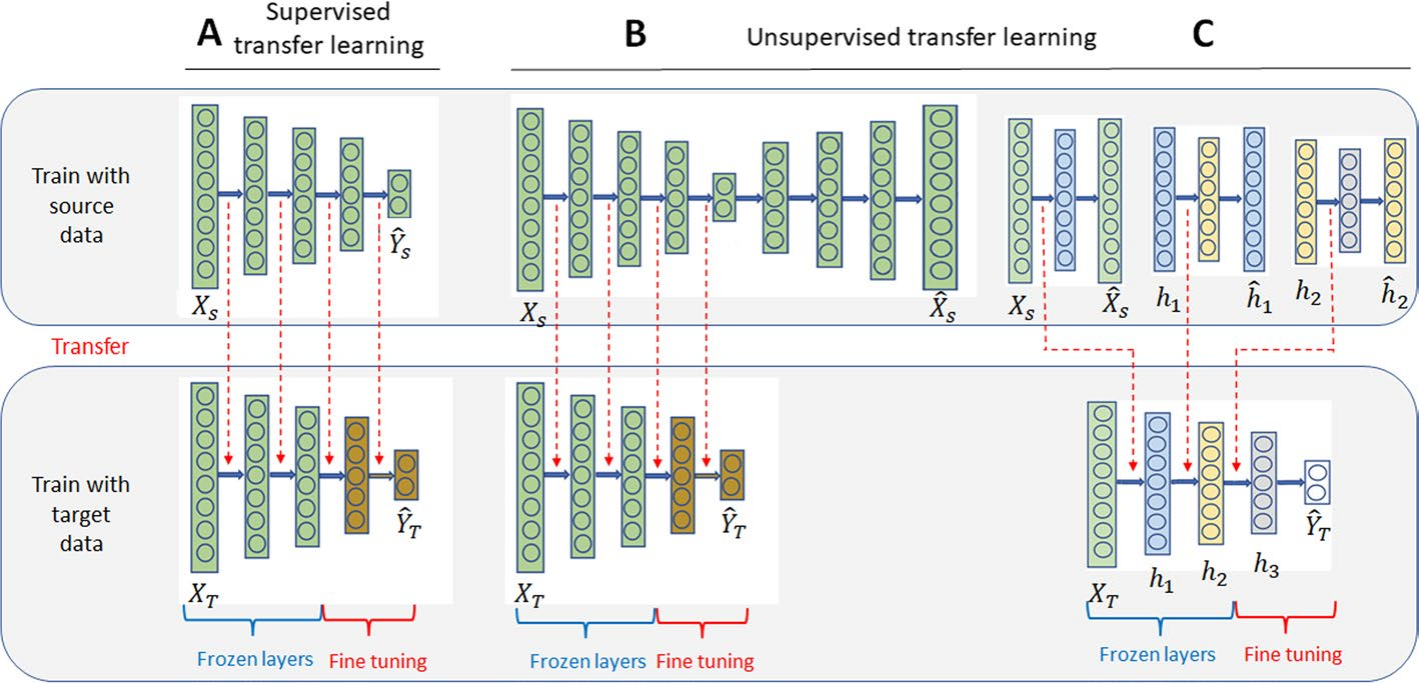
Supervised (**A**) and AE based unsupervised (**B**, **C**) transfer learning methods

In our experiments, we define different target tasks (presence of cancer or type of cancer) using the same feature space (same features) with different distributions. We apply different transfer learning approaches using the deep neural network classifier described in Sect. 5.1 as a target classifier:

- Supervised transfer learning: source domain labels are used in the construction of the source model. The NN model is first trained using the source training set. Then, the first *F* layers are frozen ($F \in \{0,\dots ,L-1 \}$), i.e., the weights of these layers are kept fixed. Finally, a second network training, called fine-tuning, is performed using a target dataset. Note that during the fine-tuning procedure, the unfrozen layers are not reinitialized, the weights from the first training step are kept (see Fig. 11A).
- Unsupervised transfer learning: labels of the source domain are not used. The hidden layers of the model are pre-trained by an AE in using the source data. The AE is a model that tries to compress the input information in the middle layer and reconstruct the input at the output. For more stability, we add Gaussian noise to the input. The objective of the model is to reconstruct the denoising input data. Its loss fonction is $Loss = \sum _{i=0}^{N} || {\mathcal {G}}(x_i+\epsilon ) - x_i ||_2$ where ${\mathcal {G}}$ is the model and $\epsilon$ is a random Gaussian noise. This model is called denoising autoencoder (DAE). There are two ways to pre-train a model with a DAE. The first one is to train a DAE whose encoder copies the architecture of the model (see Fig. 11B). The second one consists to train each hidden layer independently and successively with a DAE (see Fig. 11C). After the pre-training, the model is fine-tuned with the target data, and eventually the first *F* layers may be frozen. In our experiments, both approaches have been tested.

## Data Availability

All codes are available (https://entrepot.ibisc.univ-evry.fr/d/fd8a9fc4e00d4544ace6/). The datasets are available on the public microarray data repository ArryExpress (accession number E-MTAB-3732) and GDC data portal (TCGA).

## Abbreviations

AE: AutoEncoder
CNN: Convolutional neural network
DAE: Denoising AutoEncoder
GNN: Graph neural network
LASSO: Least Absolute Shrinkage and Selection Operator
MLP: Multi layer perceptron
NN: Neural network
RF: Random forest
SVM: Support vector machines
XGB: eXtreme Gradient Boosting
SGD: Stochastic gradient descent

## Footnotes

1 The TCGA Research Network: https://www.cancer.gov/tcga.
